# Supplementary material for: Discovery of C^2 rotation anomaly in topological crystalline insulator SrPb
Source: Nat Commun. 2021 Apr 6;12:2052. doi: 10.1038/s41467-021-22350-6 (PMC8024327; doi:10.1038/s41467-021-22350-6)
Supplement: Supplementary file 1 — Supplementary Information [file 41467_2021_22350_MOESM1_ESM.pdf]

Supplementary Information for:

Discovery of $\widehat{C}_2$ rotation anomaly in topological crystalline insulator SrPb

Wenhui Fan[1,2,*], Simin Nie[3,*], Cuixiang Wang[1,2,*], Binbin Fu[1,2], Changjiang Yi[1], Shunye Gao[1,2], Zhicheng Rao[1,2], Dayu Yan[1,2], Junzhang Ma[4,5], Ming Shi[5], Yaobo Huang[6], Youguo Shi[1,2,7,#], Zhijun Wang[1,2,#], Tian Qian[1,7,#], and Hong Ding[1,2,7]

[1] *Beijing National Laboratory for Condensed Matter Physics and Institute of Physics, Chinese Academy of Sciences, Beijing 100190, China*

[2] *University of Chinese Academy of Sciences, Beijing 100049, China*

[3] *Department of Materials Science and Engineering, Stanford University, Stanford, CA 94305, USA*

[4] *Department of Physics, City University of Hong Kong, Kowloon, Hong Kong*

[5] *Swiss Light Source, Paul Scherrer Institute, CH-5232 Villigen, PSI, Switzerland*

[6] *Shanghai Synchrotron Radiation Facility, Shanghai Advanced Research Institute, Chinese Academy of Sciences, Shanghai 201204, China*

[7] *Songshan Lake Materials Laboratory, Dongguan, Guangdong, 523808, China*

[*] These authors contributed to this work equally.

Corresponding authors: tqian@iphy.ac.cn, wzj@iphy.ac.cn, ygshi@iphy.ac.cn

Supplementary Note 1. Photon energy dependent ARPES measurements along $\bar{\Gamma}-\bar{Z}$

To determine momentum locations of the photoelectrons in the k_y direction perpendicular to the cleavage (010) surface, we have performed photon energy dependent ARPES measurements with the slit of the detector along $\bar{\Gamma}-\bar{Z}$. The inner potential V_0 is estimated to be 12 eV with lattice constants b = 12.23 Å and c = 4.648 Å in Supplementary Fig. 1.

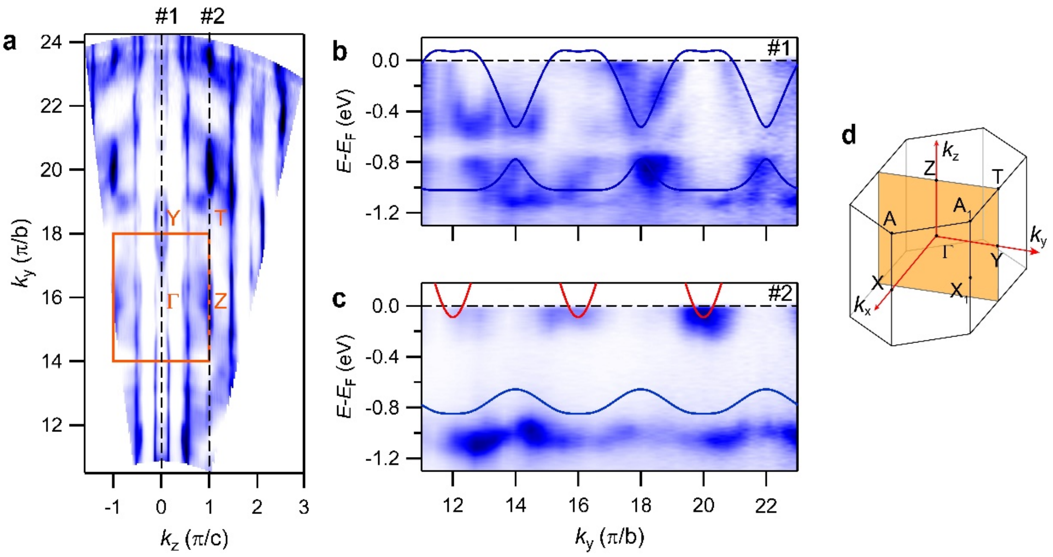

Supplementary Fig. 1. Photon energy dependent ARPES measurements along $\bar{\Gamma}-\bar{Z}$. a, ARPES intensity map at E_F measured in a photon energy range from 22 eV to 140 eV, showing the Fermi surfaces in the k_y-k_z plane. **b**,**c**, ARPES intensity plots showing the band dispersions in the k_y direction along $\Gamma-Y$ and $Z-T$, respectively. The blue and red curves represent the calculated valence and conduction bands, respectively. **d**, The yellow plane indicates the k_y-k_z plane in the bulk Brillouin zone.

Supplementary Note 2. Photon energy dependent ARPES measurements along $\bar{\Gamma} - \bar{X}$

In order to clarify band dispersions along $\bar{\Gamma} - \bar{X}$, we have performed ARPES measurements along $\bar{\Gamma} - \bar{X}$ at different photon energies between 20 eV and 40 eV. We identify two hole-like bands across E_F from the data shown in Supplementary Fig. 2. The inner band exhibits obvious variation while the outer band remains unchanged with varying photon energy. We extract the outer band dispersions at different photon energies and plot them in Fig. 4c. The conduction bands only show the bottom as two points at $k_x = \pm 0.3$ Å^{-1} in the ARPES data collected at 30 K in Supplementary Fig. 2, while the electron-like conduction bands are observed above E_F in the data at 200 K in Fig. 4d-g. Moreover, one can discern a nearly flat band at E_F between the valence and conduction bands in Supplementary Fig. 2, which is clearly revealed in the data at 200 K in Fig. 4f,g.

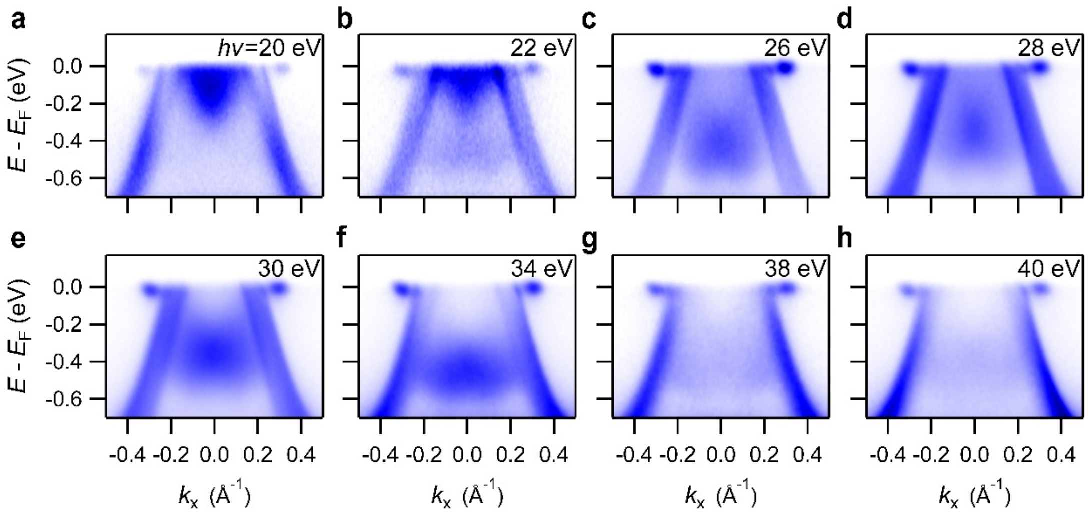

Supplementary Fig. 2. Photon energy dependent ARPES measurement along $\bar{\Gamma} - \bar{X}$. a-h, ARPES intensity plots showing band dispersions along $\bar{\Gamma} - \bar{X}$ measured with different photon energies.

Supplementary Note 3. Bulk band dispersions along $\bar{\Gamma}-\bar{X}$

In order to reveal the outline of valence bands along $\bar{\Gamma}-\bar{X}$, we have conducted second derivatives of the ARPES raw data with respect to energy. As seen in Supplementary Fig. 3b, a shoulder-like band emerges in the energy range between -0.1 eV and -0.2 eV, in good agreement with the projection of calculated bulk bands on $\bar{\Gamma}-\bar{X}$ in Fig. 2d. We note that because the second derivatives with respect to energy usually suppress the visuality of vertical bands, the outer hole-like band becomes invisible above -0.2 eV in Supplementary Fig. 3b. It can be identified in the 2D curvature intensity plot in Fig. 4g of the same raw data.

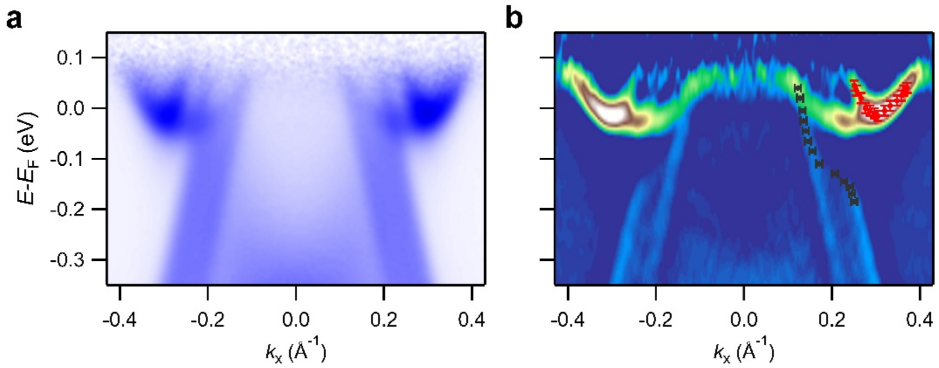

Supplementary Fig. 3. Bulk band dispersions along $\bar{\Gamma}-\bar{X}$. a, ARPES intensity plot along $\bar{\Gamma}-\bar{X}$ measured with $h\nu =$ 30 eV at 200 K. **b**, Corresponding second-derivative intensity plot. The black and red marks indicate the outlines of valence and conduction bands, respectively.

Supplementary Note 4. Calculated surface states for different terminations in SrPb

The lattice of SrPb is formed by a stacking of slightly buckled SrPb layers along the [010] direction. There are two possible cleavage planes, indicated as yellow and green planes in Supplementary Fig. 4a. For both cases, the terminations are the SrPb layer but with different dangling bonds, which are defined as terminations A and B, corresponding to cleavage planes A and B, respectively. For termination A, the cleaving breaks one chemical bond for each Sr or Pb atom, while for termination B, the cleaving breaks two chemical bonds for each Sr or Pb atom.

We conducted slab calculations for terminations A and B, whose band structures along $\bar{\Gamma}-\bar{X}$ are plotted in Supplementary Fig. 4c,d. The calculated surface states for termination A in Supplementary Fig. 4c well reproduce the experimental results in Supplementary Fig. 4b, while the calculated surface states for termination B in Supplementary Fig. 4d are inconsistent. Therefore, we infer that the real cleavage surface should be termination A.

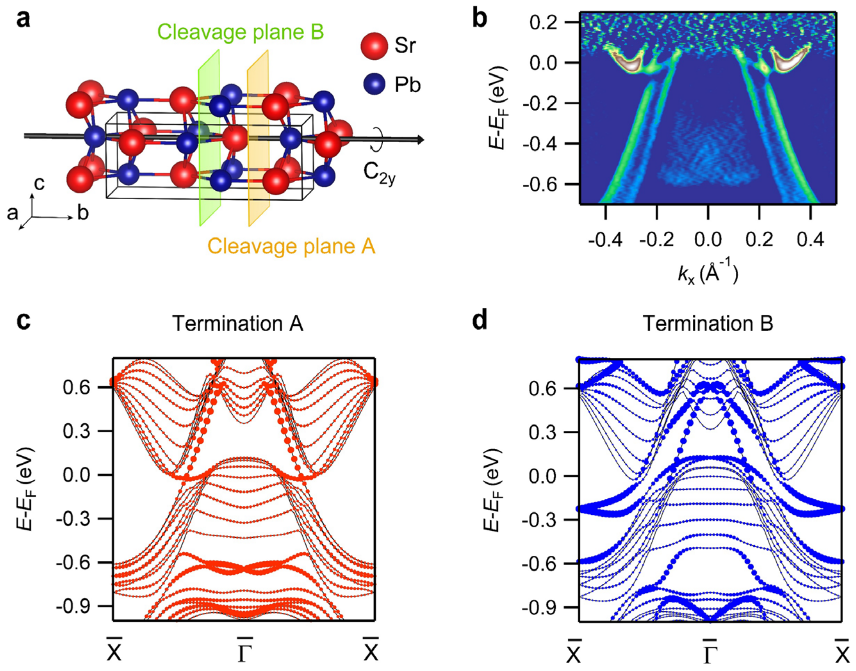

Supplementary Fig. 4. Calculated surface states for different terminations. a, Crystal structure of SrPb with two possible cleavage planes indicated as yellow and green planes. **b**, 2D curvature intensity plot showing experimental band dispersions of the Dirac surface states along $\bar{\Gamma}-\bar{X}$. **c**,**d**, Calculated band structures along $\bar{\Gamma}-\bar{X}$ of

slabs with a thickness of five unit cells along the [010] direction for terminations A and B. The size of red and blue dots scales the contribution of the outmost two SrPb layers.
